# Practical Departments

**Published:** 1902-12-13

**Authors:** 


### PRACTICAL DEPARTMENTS.

### BATHS FOR WORKMEN.

The employés of Sir Bernard Samuelson, and their wives as well, should bless that gentleman for introducing into his works at Newport, near Middlesbrough, a convenience which is an innovation in this country, although it is known in Germany and America. He has placed baths in his works so that the men can wash thoroughly before they leave the premises, and if they choose, change their clothing, and go out into the street without those marks of toil which make most of us, however much we esteem the working man in theory, keep away from him as much as possible in practice. If our working men are not cleanly, if they remain "the great unwashed," the fault it not wholly theirs. In their little homes fixed-in baths are not provided, and movable ones, besides being heavy and clumsy things to handle, take up too much room to be indulged in. And the fact that the husband comes home greasy and grimy from his work and dirties everything he touches, is a not unreasonable excuse for the wife being something of a slattern. If the man of the house comes home clean, he is more likely to expect and in time to find a clean home awaiting him. And probably the cleansing influence of the bath at the works would before long extend far beyond its direct action, and wife and children would feel themselves compelled to be clean and tidy to a far greater extent than they do now. And perhaps the fact that workmen would change their working clothes before they left the works, and would appear in street, or tram, or train, dressed like any other men, would help to throw them into more familiar contact with men engaged in other occupations, and thus help to break down that class prejudice which, whether it is avowed or not, tends to embitter the feelings of all sides when differences of opinion arise in the labour world.

